# Race, Age, and Obesity Disparities in Adult Physical Activity Levels in Breast Cancer Patients And Controls

**DOI:** 10.3389/fpubh.2014.00150

**Published:** 2014-09-19

**Authors:** Cheryl L. Thompson, Cynthia Owusu, Nora L. Nock, Li Li, Nathan A. Berger

**Affiliations:** ^1^Department of Family Medicine, Case Western Reserve University, Cleveland, OH, USA; ^2^Department of Epidemiology and Biostatistics, Case Western Reserve University, Cleveland, OH, USA; ^3^Case Comprehensive Cancer Center, Case Western Reserve University, Cleveland, OH, USA; ^4^Department of Medicine, Case Western Reserve University, Cleveland, OH, USA

**Keywords:** physical activity, breast cancer, disparities, outcomes, race, obesity

## Abstract

Physical activity has been shown to be inversely associated with breast cancer recurrence and survival. Although physical activity is known to decline with age, rates of change in physical activity have not been well characterized in breast cancer patients and subgroups with known disparities in breast cancer survival, especially in minorities, the elderly, and the obese. We evaluated moderate and strenuous physical activity from high school through diagnosis in 1,220 breast cancer patients, and from high school to recruitment in 935 controls. We compared the proportion of patients and controls meeting the American Cancer Society (ACS) guidelines for physical activity and differences in declines in level of physical activity by race, age, and obesity. At diagnosis, only 33.2% of breast cancer patients met the ACS physical activity guidelines. Only 13.2, 24.7, and 30.5% of African-American (AA), obese, and older (≥65 years) patients met the guidelines, respectively. Controls showed slightly higher rates, with 36.4% overall, 23.7% of AA, 29.0% of obese, and 32.4% of older women meeting the guidelines. AA patients were less likely to meet guidelines compared to White patients (*p* < 0.0001). Obese patients were less likely to meet guidelines compared to non-obese (*p* < 0.0001). We found that both moderate and strenuous physical activity declined after high school in patients and controls. AA patients reported steeper declines in strenuous (*p* = 0.0027), and total (*p* = 0.0009) physical activity compared to Whites. Obese patients reported steeper declines in total physical activity compared to non-obese (*p* = 0.022). Differences in average slopes of declines in physical activity were not observed by age. Our results suggest that strategies and programs to encourage women to maintain recommended levels of physical activity after high school are needed. Furthermore, breast cancer patients, particularly AA and obese patients, should be targeted to help reduce disparities.

## Introduction

Breast cancer is the most common cause of invasive cancer and the second most common cause of cancer deaths in women in the United States (US) (1). In 2014, the number of new cases is estimated to be 232,670 with 40,000 deaths expected (1). Racial disparities in breast cancer mortality have been noted since the 1980s, with African-Americans (AAs) having a lower incidence but a higher 5-year mortality compared to non-Hispanic Whites (2). Recent data indicate the age adjusted incidence of newly diagnosed breast cancer to be about 127 per 100,000 in Non-Hispanic Whites, compared to 118 per 100,000 in AAs. However, breast cancer mortality rates are estimated to be 22.7 per 100,000 for Non-Hispanic Whites and 30.8 per 100,000 for AAs (1). With earlier diagnosis and better treatments, survival has been improving in all groups of breast cancer patients. However, disparities still exist between AA and White women, with possible contributors including differences in socioeconomic status (SES), access to health care, especially screening and adjuvant therapy, and molecular and pathologic mechanisms (3–6). In a recent, large, population-based study using SEER Medicare data to examine racial differences in breast cancer survival, Silber et al. (7) found that, after accounting for the effects of demographics, clinical presentation, and treatment modalities, there remained a 4.4% 5-year survival disparity, suggesting additional factors contribute to these disparities in survival (7).

Lifestyle factors including obesity, exercise, sedentary activity, and sleep have been shown to have a profound impact on the incidence and survival of many types of cancer, including breast cancer (8–14). While these factors clearly contribute to prognosis in breast cancer patients, their potential differences have not received sufficient evaluation as a potential cause for AA–White disparities in outcomes. However, breast cancer survivors who are obese at diagnosis face poorer overall prognosis (15–18), and this disparity appears to be independent of subtype of breast cancer (19). In addition, older breast cancer patients have lower survival compared to younger patients (15).

A recent report from Europe found that decreased pre-diagnostic physical activity in newly diagnosed patients with breast cancer was associated with increased overall and breast cancer specific mortality (20). In addition, a recent report from the US showed that breast cancer survivors, in particular AA breast cancer survivors, are not meeting physical activity guidelines. We therefore sought to determine what fraction of the patients were meeting guidelines for physical activity among newly diagnosed breast cancer patients in the US, and to see if decreases in physical activity occur in association with breast cancer diagnosis, as a pre-morbid condition, or if it occurs independently over a longer time period. To evaluate the pre-diagnostic physical activity and trajectories of physical activity over time of US patients with newly diagnosed breast cancer, and to determine potential differences in groups facing disparities (AA, obese, and older), we examined moderate, strenuous, and total exercise levels in an epidemiological study of 1,220 women newly diagnosed with breast cancer and 935 women receiving a negative screening mammography (controls) to determine how many were meeting guidelines. We then assessed differences in physical activity levels and percentages meeting guidelines among groups of patients with poorer outcomes, specifically AA, older, and obese.

## Materials and Methods

### Study population and data collection

This study utilized data obtained from breast cancer patients participating in an ongoing case–control study designed to identify genetic and environmental factors of changes in mammographic density over time, breast cancer risk, and prognosis after breast cancer diagnosis (21). Breast cancer patients (*N* = 1,220) were recruited from women diagnosed with ductal carcinoma *in situ* or invasive breast cancer at University Hospitals Case Medical Center (UHCMC), which serves Cleveland and all of Northeast Ohio, from January 2007 through June 2013. Controls (*N* = 935) were recruited from women undergoing routine screening mammography at UHCMC during the same time frame. Because this population was recruited to investigate genetic and lifestyle variants associated with risk of sporadic breast cancer as well as changes in mammographic density over time, controls were eligible if they obtained at least three screening mammograms prior to enrollment and had not been diagnosed with any cancer. Breast cancer patients were eligible if they were diagnosed within the previous 3 years, obtained at least three screening mammograms prior to diagnosis, and were not known carriers of a BRCA1 or BRCA2 mutation. Of the eligible patients, 76% of cases and 69% of controls agreed to participate in the study. Those that chose not to participate did not differ with respect to age, race, or stage at diagnosis (for cases) compared to those who enrolled (*p* > 0.05).

All patients were surveyed over the phone by a trained research assistant for potential breast cancer risk factors. This survey was based on the baseline questionnaire of the California Teacher’s study (http://www.calteachersstudy.org/surveys/BaselineL.pdf), which includes questions on frequency of both self-reported moderate and strenuous (as determined by the participant) physical activity at five time points – high school, ages 18–24, 25–34, 35–44, 45–54, and the year prior to diagnosis (or the year prior to study entry, for controls). For responses that included ranges of hours per week, we took the midpoint of the range for continuous analysis. We note that this questionnaire does not ask about light intensity physical activity. For women younger than 45 (*N* = 40), physical activity during ages 45–54 years was coded as missing, and for women younger than 35 (*N* = 1), physical activity during ages 35–44 years was coded as missing. No women in this study were younger than 25 years old. Total physical activity was defined as the sum of moderate and strenuous physical activity. Age was defined as age at diagnosis for cases and age at recruitment for controls. Body mass index (BMI) was calculated based on self-reported height and weight at diagnosis for cases or recruitment for controls. Patients diagnosed at Stage 4 (*N* = 8) were excluded from this analysis. Ethical approval for this study was approved by the UHCMC Institutional Review Board. All patients provided informed written consent.

The trajectories of change from high school to current were calculated by calculating the average slope in physical activity for each individual from high school to current, using moderate, strenuous, and total (moderate plus strenuous).

To determine which groups of cancer patients and controls are more or less likely to meet established physical activity guidelines, as well as predictors of who were most likely to meet the guidelines, we used self-reported physical activity at diagnosis. Since there are different recommendations for physical activity, we chose to use the widely cited American Cancer Society (ACS guidelines) (22). Each individual was defined as meeting the ACS guidelines if she reported at least 1.25 h of strenuous activity per week or 2.5 h of combined strenuous and moderate physical activity per week. These total hours of activity per week do not depend on the number of days the activity occurred.

### Statistical analyses

Mean physical activity was calculated for all cases and controls, and also dichotomized by race (White and AA, other races were excluded from this analysis due to very small numbers), obesity (obese: current BMI ≥30 kg/m^2^; non-obese: current BMI <30 kg/m^2^) at diagnosis for cases, and at recruitment for controls, and age (younger: <65 years old; older: at least 65 years). A *t*-test was used to compare age and BMI means, and a chi-square test was used to compare race distribution between cases and controls. A *t*-test was used to assess statistically significant differences in the mean differences in physical activity hours between two comparison groups. Differences in proportions of groups of women meeting guidelines were assess via a chi-square test. A *t*-test was also used to compare differences in mean declines in physical activity between groups of study participants. In the case where equality of variances was not observed in the two groups (*p* for equality <0.05), a Satterthwaite *t*-test statistic was used instead. All missing data was treated as missing and those patients were excluded from subset analyses.

Predictors of meeting the ACS criteria for the guidelines were assessed via a multivariable unconditional logistic regression using age, race, stage at diagnosis, and BMI. Statistical significance was set at a two-sided *p*-value ≤0.05. SAS Version 9.3 (SAS Institute, Cary, NC, USA) was used for all statistical analyses.

## Results

Demographic characteristics of the breast cancer patients and controls are presented in Table 1. The control group contained a higher proportion of AA subjects (26.1 vs. 13.3%, *p* < 0.0001), but were not statistically different from cases in terms of age, BMI, or physical activity level (Table 1). Levels of moderate, strenuous, and total physical activity were similar in both breast cancer patient and control groups. There was a trend toward greater decrease from high school to the present in moderate and total physical activity among cases compared to controls; however, this did not reach statistical significance (Table 1).

**Table 1:** **Characteristics of breast cancer case and control study population**.

	Cases (*N* = 1,220)	Controls (*N* = 935)	*p*^a^
Age (years), mean (SD)	58.2 (11.4)	56.8 (10.4)	0.0022
Current BMI (kg/m^2^), mean (SD)	28.3 (6.5)	28.4 (7.0)	0.69
Race, *N*(%)			<0.0001
African-American	162 (13.3%)	243 (26.1%)	
White	1039 (85.2%)	676 (72.5%)	
Other	18 (1.5%)	14 (1.5%)	
Current moderate physical activity (h/week), mean (SD)	1.7 (2.7)	1.8 (2.9)	0.40
Current strenuous physical activity (h/week), mean (SD)	0.7 (1.9)	0.8 (2.1)	0.32
Current total^b^ physical activity (h/week), mean (SD)	2.4 (3.6)	2.6 (3.7)	0.27
Average decline in moderate physical activity since high school (h/week), mean (SD)	0.8 (3.9)	0.5 (4.0)	0.089
Average decline in strenuous physical activity since high school (h/week), mean (SD)	1.5 (3.7)	1.4 (3.8)	0.53
Average decline in total physical activity since high school (h/week), mean (SD)	2.4 (5.8)	1.9 (5.9)	0.062

*^a^*p*-Value for the univariate difference between cases and controls (*t*-test or chi-square)*.

*^b^Total physical activity is moderate plus strenuous*.

We subsequently evaluated the average amount of physical activity at diagnosis in the different demographic groups of the breast cancer patients, as well as the percentage of women in each group achieving ACS physical activity guidelines at diagnosis. Table 2 shows the average hours of physical activity at time of diagnosis in breast cancer patients for each of the groups and the percentage of each of these groups achieving the guidelines. Table 2 shows that among all 1,220 women with newly diagnosed breast cancer, the average total physical activity was 2.39 h per week, reaching 95.6% of the level recommended by the ACS. However, despite this noteworthy achievement by the group as a whole, the last column shows the disturbing result that only 33.2% of women in this study with newly diagnosed breast cancer met nationally recommended guidelines. Similarly, only 36% of White women with newly diagnosed breast cancer met recommended exercise guidelines, even though the average for this group, 2.57 h per week, slightly exceeded the guidelines. This is due to a skewed distribution with a relatively small number of women who greatly exceeded the recommended guidelines, among the White women in our sample, 16.8% reported at least 5 h of physical activity per week, twice the recommended minimum. In contrast, AA women with newly diagnosed breast cancer, as a group, showed an average exercise time of 1.18 h per week or only 47% of the recommended 2.5 h per week. Moreover, only 13.2% of these women achieved national guidelines. Further, older women, ≥65, averaged fewer hours, 2.2 h per week, compared to younger, <65, 2.56 h per week and a smaller percentage, 30.5%, of older women compared to 34.3% of younger women achieved recommended guidelines. Obese patients on average achieved only 1.69 h, 67% of the recommended hours. The goal of 2.5 total hours per week or 1.25 h of strenuous activity was achieved by only 27.7% of obese patients. Patients who fell into more than one of these lower physical activity groups were even less likely to meet guidelines (Table 2). Patients who were both older and AA only met the guidelines 6.4% of the time and only 5.0% of the patients who were older, obese, and AA met the guidelines.

**Table 2:** **Self-reported average hours of physical activity at diagnosis in breast cancer patients**.

Activity type	Strenuous	Moderate	Total^a^	Percent women meeting ACS Guidelines^b^ (%)
Recommended guidelines	1.25 h/week	2.5 h/week	2.5 h/week	
	Mean (SD)	Mean (SD)	Mean (SD)	
Everyone (*N* = 1,220)	0.68 (1.87)	1.74 (2.74)	2.39 (3.61)	33.2
Whites (*N* = 1,039)	0.74 (1.94)	1.84 (2.84)	2.57 (3.73)	36.0
African-Americans (*N* = 162)	0.33 (1.39)	0.84 (1.79)	1.18 (2.43)	13.2
Age <65 years (*N* = 840)	0.85 (2.14)	1.70 (2.73)	2.55 (3.81)	34.3
Age ≥65 years (*N* = 374)	0.30 (0.92)	1.73 (2.77)	2.02 (3.08)	30.5
Not obese (BMI <30) (*N* = 820)	0.83 (2.09)	1.89 (2.89)	2.72 (3.85)	37.3
Obese (BMI ≥30) (*N* = 391)	0.36 (1.25)	1.34 (2.37)	1.69 (2.94)	24.7
Obese and age ≥65 years (*N* = 125)	0.30 (0.98)	1.40 (2.47)	1.67 (2.90)	25.8
Obese and African-American (*N* = 86)	0.19 (1.00)	0.76 (1.45)	0.96 (1.80)	11.8
African-American and age ≥65 years (*N* = 47)	0.06 (0.22)	0.85 (1.57)	0.91 (1.66)	6.4
Obese, African-American, and age ≥65 years (*N* = 20)	0.06 (0.22)	0.88 (1.44)	0.93 (1.64)	5.0

*^a^Total physical activity is strenuous plus moderate*.

*^b^Recommended physical activity by the American Cancer Society (http://www.cancer.org) is 1.25 h/week of strenuous exercise or 2.5 h of total moderate and strenuous exercise, with a minimum of 2.5 h/week*.

Table 3 shows the results of the multivariable logistic regression for predictors of meeting the ACS guidelines. In this multivariable analysis, each kilograms per square meter increases in BMI was associated with a 4% reduction in likelihood of meeting the ACS guidelines (OR (per kg/m^2^) = 0.96, 95% CI = 0.94–0.99, *p* = 0.0011). This means that for a 5 kg/m^2^ increase in BMI, equivalent to going from normal weight to overweight, or overweight to obese, is associated with an approximately 20% reduction in likelihood of meeting ACS guidelines. Race was a strong predictor of meeting the guidelines such that, compared to AAs, Caucasians were over three times as likely to not meet the ACS guidelines (OR = 3.17, 95% CI = 1.92–5.21, *p* < 0.0001) (Table 3). Neither age (*p* = 0.082) nor stage at diagnosis (*p* = 0.31) was statistically significant in the multivariable analyses for likelihood of reaching the ACS physical activity guidelines.

**Table 3:** **Predictors of meeting the American Cancer Society Guidelines for physical activity**.

Variable	OR (95% CI)	*p*
Age (years)	0.99 (0.98–1.00)	0.082
Race (Caucasian vs. African-American)	3.17 (1.92–5.21)	<0.0001
Stage (compared to stage 0)
1	1.13 (0.80–1.61)	0.49
2	1.01 (0.67–1.48)	0.97
3	0.69 (0.40–1.22)	0.20
Body mass index (kg/m^2^)	0.96 (0.94–0.99)^a^	0.0011

*Results of multivariable logistic regression*.

*^a^Body mass index treated as continuous, and this odds ratio is for each unit increase in body mass index. Thus, for a 5 kg/m^2^ increase in BMI is associated with an approximately 20% reduction in likelihood of meeting ACS guidelines*.

Table 4 shows that although physical activity among control women was slightly greater than patients with newly diagnosed breast cancer, they were similar among the different groups. Importantly, even among controls, AA, and obese women showed the lowest percentage achieving national goals. Similar to patients, control women who fell into more than one of the groups with poorer outcomes were even less likely to meet guidelines. Compared to the population as a whole, for which 36.4% of the women met the guidelines, only 18.5% of those women who were at least 65 years old, obese, and AA met the guidelines.

**Table 4:** **Self-reported average hours of physical activity at recruitment among controls**.

Activity type and recommended guidelines	Strenuous	Moderate	Total^a^	Percent women meeting ACS guidelines^b^ (%)
Recommended guidelines	1.25 h/week	2.5 h/week	2.5 h/week	
	Mean (SD)	Mean (SD)	Mean (SD)	
Everyone (*N* = 939)	0.76 (2.07)	1.81 (2.86)	2.56 (3.65)	36.4
Whites (*N* = 676)	0.90 (2.24)	2.05 (3.06)	2.94 (3.99)	40.9
African-Americans (*N* = 243)	0.41 (1.40)	1.11 (2.00)	1.52 (2.62)	23.7
Age <65 years (*N* = 709)	0.88 (2.21)	1.85 (2.91)	2.71 (3.77)	37.7
Age ≥65 years (*N* = 214)	0.34 (1.23)	1.66 (2.62)	2.00 (2.84)	32.4
Not obese (BMI <30) (*N* = 628)	0.90 (2.20)	1.99 (3.04)	2.86 (3.85)	39.7
Obese (BMI ≥30) (*N* = 301)	0.49 (1.73)	1.41 (2.32)	1.90 (3.06)	29.0
Obese and age ≥65 years (*N* = 58)	0.35 (1.65)	1.09 (1.52)	1.44 (2.08)	24.1
Obese and African-American (*N* = 125)	0.46 (1.65)	1.05 (1.73)	1.52 (2.64)	24.0
African-American and age ≥65 years (*N* = 61)	0.14 (0.53)	1.33 (2.05)	1.47 (2.17)	24.6
Obese, African-American, and age ≥65 years (*N* = 27)	0.15 (0.60)	1.19 (1.89)	1.34 (1.89)	18.5

*^a^ Total physical activity is strenuous plus moderate*.

*^b^ Recommended physical activity by the American Cancer Society (http://www.cancer.org) is 1.25 h/week of strenuous exercise or 2.5 h of total moderate and strenuous exercise, with a minimum of 2.5 h/week*.

**Figure 1:**
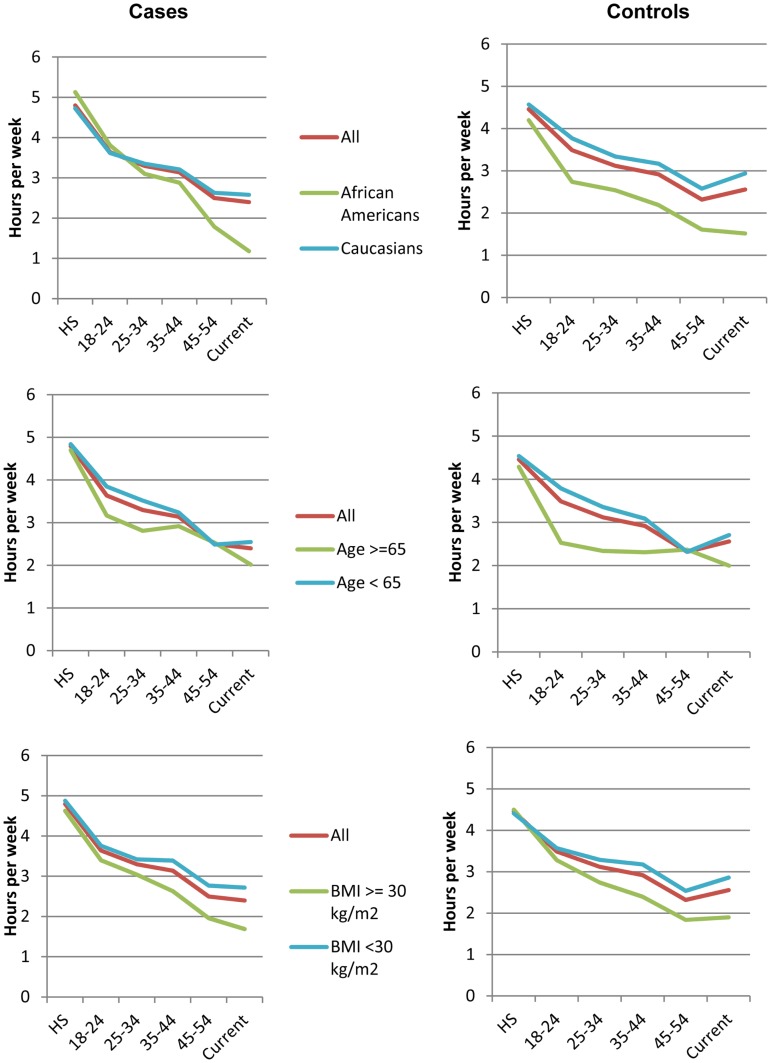
**Trajectories of physical activity levels**. Average hours of total strenuous and moderate physical activity at different ages for all breast cancer patients (left) and controls (right), including subsets of these women

We next examined average physical activity levels in high school and the trajectory of change from high school to the time of breast cancer diagnosis for cases or to time of recruitment for controls. Figure 1 illustrates the average amount of physical activity for each group of women over time. On average, patients who developed breast cancer reported 2.21 h (SD = 3.53) of strenuous and 2.53 h (SD = 3.55) of moderate activity per week during high school. Thus, on average, during high school, women well exceeded the recommended guidelines for total hours of exercise. In contrast, at the time of diagnosis, breast cancer patients reported only 0.68 h (SD = 1.87) of strenuous and 1.70 h (SD = 2.74) of moderate physical activity per week, which represents a decline of 33 and 69% in moderate and strenuous physical activity, respectively, from high school. The average hours of activity were not statistically significantly different between breast cancer patients and controls at any time point (*p* > 0.05). Overall, women in *all* subgroups showed a steady decline in physical activity between high school and time of diagnosis (Figure 1), despite starting out at high levels of moderate and strenuous activity in high school. These declines were not statistically significantly different between breast cancer patients and controls.

## Discussion

In this study, we found that declines in moderate and strenuous physical activity from high school throughout adulthood are common among all women, such that, at the time of diagnosis, 2/3 of breast cancer patients are not meeting ACS recommended guidelines. When repeating these analyses using the American College of Sports Medicine recommended guidelines of 2.5 h of total moderate and strenuous physical activity (irrespective of the proportion of strenuous), very similar results were found (data not shown). We identified steeper declines resulting in lower levels of physical activity in demographic groups with poorer breast cancer outcomes, specifically AAs and obese patients. These differential rates of decline could, in part, contribute to the recent observation that all breast cancer survivors, but especially AA survivors, as a group, do not meet ACS recommended guidelines after diagnosis (23). Importantly, our studies show that this is a problem of a gradual decline starting from time of leaving high school and that it does not occur immediately pre-morbid, co-morbid, or associated with new onset of breast cancer. Recent results from Europe also demonstrate a decline in physical activity and exercise in women following high school and lower levels in patients with breast cancer (24). Our report now demonstrates the high prevalence of this problem in the United States as well. More importantly, our studies indicate that, in the US, the decrease in exercise from high school onward is a particular problem in AA and obese women.

Although exercise has been shown to be feasible in breast cancer patients and to have salutary effects, including improvement in quality of life, increased adherence to therapeutic regimens and improved survival (25–28), the low levels of physical activity shown by both cases and controls as well as the continuous decrease from high school on, suggests a compelling need to promote regular exercise programs and their associated benefits to all women from the time they leave high school, into and throughout their adulthood. Our data further suggest the need for clinical trials to determine whether increased exercise will contribute to reducing disparities in breast cancer outcomes among these groups of patients with established poorer outcomes. Moreover, it is important to increase physical activity following diagnosis of breast cancer, since, even in patient populations that had been sedentary prior to diagnosis, increased activity has been shown to improve outcomes. In particular, Bernstein et al. (29) showed that lifetime recreational activity was associated with a reduced risk of breast cancer in a large study of both White and AA women, and that the protective effect was similar in both racial groups. It is also noteworthy that while recent studies have demonstrated the feasibility and benefits of physical activity, including both aerobic and resistance exercise, interventions in recently diagnosed breast cancer patients (26, 28, 30–34). Most of these studies have focused on White and non-elderly patients.

Our study has a number of strengths and limitations. The major strengths include the large samples size and available data from high school through diagnosis or time of recruitment. The weaknesses include the self-reported nature of the physical activity included in this study as well as the retrospective nature of this study, which could lend itself to potential biases in the recall, particularly at times such as during high school, which the average woman in our study had to recall after several decades. While most patients in the study were recruited within 6 months of diagnosis, patients remained eligible for recruitment up to 3 years following diagnosis. To see if this was likely to bias recall of physical activity, we evaluated average self-reported physical activity among the patients recruited within 6 months (*N* = 631) compared to those recruited more than 2 years post diagnosis (*N* = 160). They did not differ in their self-reported moderate (*p* = 0.062), strenuous (*p* = 0.67), or total physical activity (*p* = 0.87). In addition, we chose to use a simple slope of the change in physical activity from high school to diagnosis for cases and recruitment for controls. We felt that this change in physical activity is readily interpretable as the reduction of average hours of physical activity per week. There are other ways to analyze differences in trajectories of change over time, including fitting splines and non-linear trends, which may show differences in trajectories of change at various time points during the patients adulthood that might be of interest as well, and should be explored in future studies.

Since physical activity is a modifiable lifestyle behavior, it is critical that oncologists and institutions treating patients with breast cancer develop strategies to encourage patients to increase their activity levels, and also that facilities and trained personnel are available to guide and support these patients. This is particularly important for AA and obese patients, as these patients, on average, both have the lowest physical activity levels and the least favorable prognosis. In fact, increased physical activity in AAs who have lowest levels of physical activity or worst prognosis might be able to contribute significant improvements in prognosis and thus reduce current disparities.

## Conclusion

In conclusion, our results show that both breast cancer patients and US women in general, have significantly reduced their strenuous and moderate physical activity since high school. These declines are particularly pronounced among AAs and obese women, and newly diagnosed breast cancer patients in these groups are less likely to meet ACS physical activity guidelines. Since these are also patient populations with disparities in outcomes, increasing physical activity among these women may help reduce disparities and improve prognosis in these subgroups. The observation that physical activity declines gradually from high school in controls as well as in breast cancer patients suggests that strategies and programs to help maintain recommended physical activity levels after high school in all women are needed.

## Author Contributions

Cheryl L. Thompson is co-PI of study population from which the samples were drawn, assisted with the development of the study design, performed all statistical analyses and led the manuscript preparation. Cynthia Owusu and Nora L. Nock contributed to the study design. Li Li is co-PI of study population from which the samples were drawn. Nathan A. Berger developed the initial study design. All authors assisted with critical manuscript development and review and approved the final manuscript.

## Conflict of Interest Statement

The authors declare that the research was conducted in the absence of any commercial or financial relationships that could be construed as a potential conflict of interest.

## Abbreviations

ACS, American Cancer Society; BMI, body mass index; US, United States.
